# Synergistic cytotoxicity of gemcitabine, clofarabine and edelfosine in lymphoma cell lines

**DOI:** 10.1038/bcj.2013.69

**Published:** 2014-01-10

**Authors:** B C Valdez, A R Zander, G Song, D Murray, Y Nieto, Y Li, R E Champlin, B S Andersson

**Affiliations:** 1 Department of Stem Cell Transplantation and Cellular Therapy, University of Texas MD Anderson Cancer Center, Houston, TX, USA; 2 Department of Stem Cell Transplantation, University Medical Center Hamburg-Eppendorf, Hamburg, Germany; 3 Department of Experimental Oncology, Cross Cancer Institute, Edmonton, Alberta, Canada

**Keywords:** edelfosine, gemcitabine, clofarabine, synergism, apoptosis, lymphoma

## Abstract

Treatments for lymphomas include gemcitabine (Gem) and clofarabine (Clo) which inhibit DNA synthesis. To improve their cytotoxicity, we studied their synergism with the alkyl phospholipid edelfosine (Ed). Exposure of the J45.01 and SUP-T1 (T-cell) and the OCI-LY10 (B-cell) lymphoma cell lines to IC_10_–IC_20_ levels of the drugs resulted in strong synergistic cytotoxicity for the 3-drug combination based on various assays of cell proliferation and apoptosis. Cell death correlated with increased phosphorylation of histone 2AX and KAP1, decreased mitochondrial transmembrane potential, increased production of reactive oxygen species and release of pro-apoptotic factors. Caspase 8-negative I9.2 cells were considerably more resistant to [Gem+Clo+Ed] than caspase 8-positive cells. In all three cell lines [Gem+Clo+Ed] decreased the level of phosphorylation of the pro-survival protein AKT and activated the stress-activated protein kinase/c-Jun N-terminal kinase (SAPK/JNK) stress signaling pathway, which in J45.01 cells resulted in the phosphorylation and heterodimerization of the transcription factors ATF2 and c-Jun. The observed rational mechanism-based efficacy of [Gem+Clo+Ed] based on the synergistic convergence of several pro-death and anti-apoptotic signaling pathways in three very different cell backgrounds provides a powerful foundation for undertaking clinical trials of this drug combination for the treatment of lymphomas.

## Introduction

Lymphomas are hematological abnormalities of the B or T lymphocytes that may develop in the lymph nodes, spleen, bone marrow and blood. Treatment options include chemotherapy, radiation therapy, hematopoietic stem-cell transplantation or their combination. Due to the heterogeneity of the disease, efficacious lymphoma-specific treatment regimens need to be developed.

In our continued search for more efficacious and safer drug combinations for hematologic malignancies, we recently reported the synergistic cytotoxicity of gemcitabine (Gem) and clofarabine (Clo) in multiple myeloma cell lines and primary cells derived from patients with multiple myeloma.^1^ The observed synergism of these two nucleoside analogs could be partly attributed to Gem-mediated activation/phosphorylation of deoxycytidine kinase, inhibition of DNA synthesis and DNA repair, nucleolar stress through inhibition of rRNA production and induction of apoptosis.^1^ These nucleoside analogs are not as cytotoxic in lymphoma cell lines, and we hypothesized that [Gem+Clo] cytotoxicity might be enhanced by combination with drug(s) with different mechanism(s) of action. An example of such an anti-neoplastic drug is edelfosine (1-O-octadecyl-2-O-methyl-rac-glycero-3-phosphocholine, ET-18-O-CH3), which belongs to a new class of alkyl phospholipids with *in vitro* anti-neoplastic activity in non-Hodgkin lymphoma, leukemia, breast cancer, pancreatic cancer and prostate cancer.^2, 3, 4, 5, 6, 7^ Edelfosine (Ed) was evaluated in phase I and II studies where it demonstrated activity in non-small cell lung cancer and glioblastoma multiforme with very few adverse events, mainly gastrointestinal toxicity.^8, 9^

Edelfosine mainly incorporates into lipid rafts in the cell membrane, changes their organization and activates the Fas/CD95 cell-death receptor.^10^ It inhibits the MAPK/ERK mitogenic pathway and the AKT/protein kinase B survival pathway.^11, 12, 13, 14^ Among its subcellular targets are endoplasmic reticulum and mitochondria.^15, 16^

To evaluate the possibility of using the combination of [Gem+Clo] and Ed for lymphoma patients, we used lymphoma cell line models to study their cytotoxicity and mechanisms of action. Using concentrations close to their IC_10_–IC_20_ values, combination of the three drugs showed strong synergistic cytotoxicity. We attribute this synergism to two different death signaling pathways that initiate from the cell membrane (for ED) and nucleus (for Gem and Clo) and centrally converge on the mitochondria. Moreover, [Gem+Clo+Ed] activates the stress-activated protein kinase/c-Jun N-terminal kinase (SAPK/JNK) stress-signaling pathway through ATF2 and consequently elicits cancer cell death.

## Materials and methods

### Cell lines and drugs

The J45.01 and SUP-T1 T-cell lymphoma cell lines were obtained from the American Type Culture Collection (Manassas, VA, USA). J45.01 is a CD45-deficient variant of Jurkat, a human T-cell lymphoblast line that was originally established from a patient with T-cell leukemia and is now widely used as a cellular model for T-cell lymphoma. The OCI-LY10 B-cell lymphoma cell line was kindly provided by Dr Richard J Ford, Jr (UT MD Anderson Cancer Center, Houston, TX, USA). Cells were cultured in RPMI-1640 (Mediatech, Manassas, VA, USA) supplemented with 10% heat-inactivated fetal bovine serum (Sigma-Aldrich, St Louis, MO, USA) and 100 U/ml penicillin and 100 μg/ml streptomycin (Mediatech) at 37 °C in a humidified atmosphere of 5% CO_2_. Edelfosine was obtained from Medmark Pharma GmbH (Munich, Germany) and a 10 mg/ml stock solution was freshly prepared in ethanol; it was further diluted with RPMI-1640 medium. Clofarabine (Clolar) was purchased from Genzyme Oncology, Cambridge, MA, USA (1 mg/ml solution) and diluted in RPMI-1640 medium before use, and gemcitabine (Eli Lilly, Indianapolis, IN, USA) was dissolved in phosphate-buffered saline (PBS).

### Cytotoxicity assay

Cell suspensions were aliquoted (100 μl of 2 × 10^5^ cells/ml) into 96-well plates in the presence of drug(s) or solvent alone and continuously incubated at 37 °C for 48 h. The cells were analyzed for cytotoxicity by the 3-(4,5-dimethylthiazol-2-yl)-2,5-diphenyl tetrazolium bromide (MTT) assay.^17^ Briefly, 50 μl of 5 μg/μl MTT reagent in PBS was added per well and incubated for 4 h at 37 °C. The solid reaction product was dissolved by adding 100 μl solubilization solution (0.1 N HCl in isopropanol containing 10% Triton X) to each well, mixing, and incubating at 37 °C overnight. Absorbance at 570 nm was measured using a Victor X3 (Perkin Elmer Life and Analytical Sciences, Shelton, CT, USA) plate reader. Proliferation was determined relative to the control cells exposed to solvent alone. Graphical analyses including calculations of IC_10_ –IC_20_ values (the concentration of drug required for 10–20% growth inhibition) were done using Prism 5 (GraphPad Software, San Diego, CA, USA). Drug combination effects were estimated based on the combination index (CI) values^18^ calculated using the CalcuSyn software (Biosoft, Ferguson, MO, USA).

### Apoptosis assay

Cells were exposed to drugs for 48 h and analyzed for apoptosis by flow-cytometric measurements of phosphatidylserine externalization with Annexin-V-FLUOS (Roche Diagnostics, Indianapolis, IN, USA) and 7-aminoactinomycin D (BD Biosciences, San Jose, CA, USA) using a Muse Cell Analyzer (EMD Millipore, Billerica, MA, USA). The extent of cleavage of poly(ADP-ribose) polymerase (PARP) -1 and caspases 3 and 8, determined by western blotting, was also used as an indicator of apoptosis.

### Western blot analysis

Cells exposed to drugs or solvent for 48 h were collected by centrifugation, washed with ice-cold PBS and lysed with cell lysis buffer (Cell Signaling Technology, Danvers, MA, USA). The protein concentrations were determined using a BCA Protein Assay kit (ThermoFisher Scientific, Inc., Rockford, IL, USA). Proteins were resolved on polyacrylamide-SDS gels and blotted onto nitrocellulose membranes (Bio-Rad, Hercules, CA, USA). Western blot analyses were done by chemiluminescence using the Immobilon Western Chemiluminescent HRP Substrate (EMD Millipore). Most of the antibodies, their sources and other relevant information were previously described.^1, 19, 20^ Antibodies against apoptosis-inducing factor, AKT, p-AKT (Ser473), p-AKT (Thr308), ATF2, p-ATF2 (Thr71), Bax, cleaved caspase 8, c-Jun, p-c-Jun (Ser73), COX 4, cytochrome *c*, 4E-BP1, p-4E-BP1 (Thr37/46), PDK1, p-PDK1 (Ser241), SAPK/JNK and p-SAPK/JNK (Thr183/Tyr185) were purchased from Cell Signaling Technology. Antibodies against p16 and ANP32B proteins were obtained from Santa Cruz Biotechnology, Inc. (Dallas, TX, USA) and Proteintech Group, Inc. (Chicago, IL, USA), respectively.

### Analysis of reactive oxygen species

J45.01 cells exposed to drug(s) for 24 h were analyzed for early production of reactive oxygen species (ROS) using CM-H_2_DCFDA (5-(and-6)-chloromethyl-2′,7′-dichlorodihydrofluorescein diacetate, acetyl ester), an ROS indicator that diffuses into cells where it is oxidized to a fluorescent product (Life Technologies, Grand Island, NY, USA). Briefly, cells were aliquoted (0.5 ml) into 5 ml tubes and 1 μl of 1.5 mM CM-H_2_DCFDA (dissolved in dimethyl sulfoxide) was added. Cells were incubated at 37 °C for 1 h and immediately analyzed with a Gallios Flow Cytometer (Beckman Coulter, Inc., Brea, CA, USA) using excitation/emission wavelengths of 492/520 nm. Geometric means of the fluorescence intensities were compared and the relative fold increase in ROS production was calculated.

### Analysis of mitochondrial membrane potential

A mitochondrial membrane potential detection kit (Cayman Chemical Co., Ann Arbor, MI, USA) was used to determine changes in the mitochondrial membrane potential (Δψm) using the JC-1 (5,5′,6,6′-tetrachloro-1,1′,3,3′-tetraethylbenzimidazolylcarbocyanine iodide) reagent. J45.01 cells to be analyzed were aliquoted (0.5 ml) into 5 ml tubes. Valinomycin (1 μM) was added to a positive control tube containing untreated cells and incubated at 37 °C, 5% CO_2_ for 15 min. Diluted (1:10 with cell growth medium, 40 μl) Δψm-sensitive fluorescent dye JC-1 reagent was added to each tube, incubated at 37 °C for 20 min, and immediately analyzed by flow cytometry as described by the manufacturer.

### Isolation of cytosolic and mitochondrial fractions

J45.01 cells were collected by centrifugation, washed with ice-cold PBS, resuspended in buffer A (10 mM HEPES (pH 7.6), 10 mM KCl, 100 μM EDTA, 100 μM EGTA, 1 mM DTT, 500 μM phenylmethanesulfonyl fluoride and proteinase inhibitor cocktail) and incubated on ice for 30 min. Cells were lysed by passing the cells 10 × through a fine needle (27½ gauge). To separate the nuclei, cell lysates were centrifuged at 800 *g* for 5 min at 4 °C. The supernatant containing mitochondria and cytosolic extracts was further centrifuged at 12 500 *g* at 4 °C for 8 min to pellet mitochondria and the resulting supernatant was saved as the cytosolic fraction. The mitochondria in the pellet were washed with ice-cold buffer A, and resuspended in ice-cold lysis buffer B (10 mM HEPES (pH 7.6), 300 mM KCl, 1 mM EDTA, 0.5% Triton X-100, 5% glycerol, 1 mM DTT and proteinase inhibitor cocktail). The cytosolic and mitochondrial extracts were further centrifuged at 15 000 *g* for 20 min at 4 °C, and the resulting supernatants analyzed by western blotting.

### Immunoprecipitation assay

J45.01 cells were exposed to solvent or [Gem+Clo+Ed] combination for 48 h, harvested and lysed using lysis buffer (Cell Signaling Technology) supplemented with 1 mM phenylmethanesulfonyl fluoride for 1 h on ice. Cell lysates were centrifuged at 15 000 *g* for 10 min at 4 °C and the protein concentration of the supernatant was determined as described above. Approximately 400 μg total protein was diluted with ice-cold PBS containing protease inhibitors (Roche Applied Science, Indianapolis, IN, USA) to 500 μl and mixed with 50 μl (50% slurry) of pre-washed Pierce Protein A/G agarose beads (ThermoFisher Scientific, Inc.). The mixture was tumbled for 10 min at 4 °C and centrifuged at 14 000 *g* for 10 min to eliminate non-specific binding species. The supernatant was mixed with 50 ng normal rabbit IgG (Santa Cruz Biotechnology, Inc.) or 50 ng anti-ATF2 antibody (Cell Signaling Technology) and tumbled overnight at 4 °C. The mixture was centrifuged at 14 000 *g* for 10 min and the supernatant was mixed with 50 μl (50% slurry) of pre-washed Pierce Protein A/G agarose beads, tumbled for 2 h at 4 °C, centrifuged again, and the beads were washed two times before boiling in gel loading buffer (Cell Signaling Technology). Immunoprecipitated proteins were analyzed by western blotting as described above.

### Statistical analysis

Results are presented as the mean±standard deviation of four independent experiments and statistical analysis was performed using a Student's paired *t*-test with a two-tailed distribution.

## Results

### Gemcitabine, clofarabine and Ed have synergistic cytotoxicity toward lymphoma cell lines

**Figure 1:**
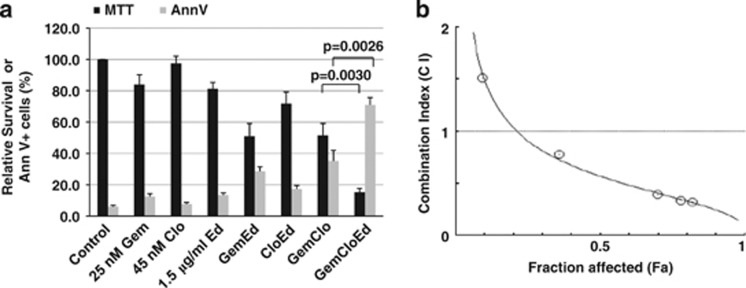
Synergistic cytotoxicity of Gem, Clo and Ed. (**a**) J45.01 cells were continuously exposed to drugs alone, or in combination, for 48 h and analyzed by the MTT assay for cell proliferation and Annexin V assay (Ann V) for early cell death. Statistically significant differences for [Gem+Clo] versus [Gem+Clo+Ed] are indicated by the *P*-values. (**b**) Cells were exposed to various concentrations of the drugs (constant ratio) for 48 h and early cell death was analyzed by Annexin V assay. The obtained fractions of dead cells (Fa) for the individual drugs and three-drug combination were used to calculate the CIs and to determine the synergism of Gem, Clo and Ed using the Chou and Talalay method.^18^ The calculated CI values for the experimental Fa for the three-drug combination relative to the individual drugs are shown. CI values less than 1 suggest synergism.

Cells were initially exposed to individual drugs and analyzed for cell survival by MTT assay. On the basis of the calculated IC_10_–IC_20_ values, we exposed J45.01 cells to 25 nM Gem, 45 nM Clo and 1.5 μg/ml Ed individually, or in various combinations, for 48 h. Exposure of J45.01 cells to a single drug inhibited cell survival by 3–19%, two-drug combinations by 28–49%, and the three-drug combination by 85% (Figure 1a). While [Gem+Clo] combination inhibited cell survival by 49%, addition of Ed to these two nucleoside analogs significantly increased inhibition to 85% (*P*=0.003). These results are consistent with the Annexin V assay, which was used to measure early cell death. Exposure of J45.01 cells to each individual drug for 48 h resulted in 8–13% Annexin V-positive cells (solvent control has 6%). The three two-drug combinations resulted in 17–35% Annexin V-positive cells, whereas the [Gem+Clo+Ed] combination resulted in 71% Annexin V-positive cells, indicating a significant cell death with the three-drug combination (Figure 1a).

To determine whether the cytotoxicity of the three drugs reflected a synergistic interaction, J45.01 cells were exposed to individual drugs or to various three-drug combinations at a constant concentration ratio, and indication of cell death was analyzed by Annexin V assay. The CIs were calculated using the CompuSyn software developed by T-C Chou and N Martin, which was based on a previously described methodology.^18^ Figure 1b shows increasing synergism (CI<1) with increasing drug effects (Fa); at 50% Annexin V-positive cells (Fa=0.5), the calculated CI=0.57 suggested strong synergistic cytotoxicity. Similar drug synergism (that is, CI<1) was seen with the SUP-T1 and OCI-LY10 lymphoma cell lines (data not shown).

### The combinations of [Gem+Clo+Ed] activate the DNA-damage response and apoptosis pathways

**Figure 2:**
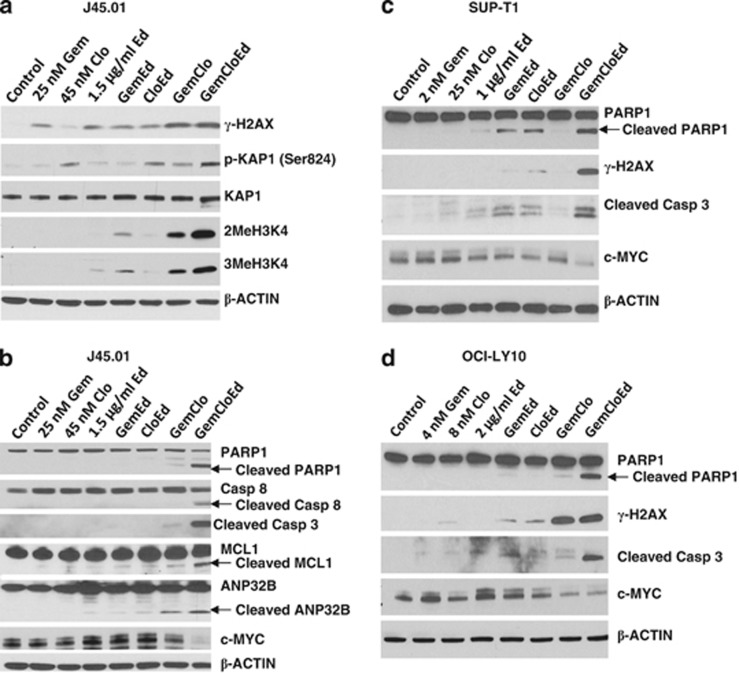
Activation of elements of the DNA-damage response (**a**) and apoptosis (**b–d**) pathways by Gem, Clo and Ed singly or in various combinations. Different lymphoma cell lines were continuously exposed to drugs for 48 h and total protein extracts were analyzed for changes in the level or modification of the indicated proteins.

Nucleoside analogs have been shown to stall replication forks when incorporated into nascent DNA strands during replication and cause DNA strand breaks.^21^ On the other hand, alkyl phospholipids cause DNA damage through production of ROS.^22^ We were thus prompted to determine whether Ed could enhance DNA damage mediated by [Gem+Clo]. A widely used indicator of the DNA-damage response is the phosphorylation of histone 2AX.^23^ Exposure of J45.01 cells to Gem, Clo or Ed alone slightly increased the level of γ-H2AX (Figure 2a), consistent with previous reports.^21, 22^ The [Gem+Clo] combination dramatically increased γ-H2AX, and this effect was slightly enhanced when Ed was added (Figure 2a).

Another indicator of the DNA-damage response is the post-translational modification of the chromatin-associated protein KAP1, which is phosphorylated by ATM after DNA damage to alter chromatin structure and allow access for the DNA-repair machinery.^24^ Although Clo, [Ed+Clo] and [Gem+Clo] stimulated the phosphorylation of KAP1, the combination of [Gem+Clo+Ed] resulted in the highest level of phosphorylation (Figure 2a). Such modification correlates with increased methylation of histone 3, further suggesting chromatin restructuring (Figure 2a).

The observed DNA-damage response and chromatin remodeling are indicative of genomic injury that may lead to apoptosis. Indeed, exposure of J45.01 cells to [Gem+Clo+Ed] combination resulted in the extensive cleavage of PARP1, activation (by cleavage) of the key pro-apoptotic proteins caspases 3/8 and inactivation (by cleavage) of the anti-apoptotic protein MCL1 (Figure 2b). The activation of caspase 3 correlates with the cleavage of one of its known substrates, ANP32B (Figure 2b), a histone chaperone.^25, 26^

Similar activation of apoptosis was observed in other lymphoma cell lines. Exposure of SUP-T1 (Figure 2c) and OCI-LY10 (Figure 2d) cell lines to [Gem+Clo+Ed] resulted in increased cleavage of PARP1 and caspase 3, increased phosphorylation of H2AX and decreased levels of the pro-survival c-MYC protein. Overall, the results suggest synergistic cytotoxicity of [Gem+Clo+Ed] in lymphoma cells of both B- and T-cell origins.

**Figure 3:**
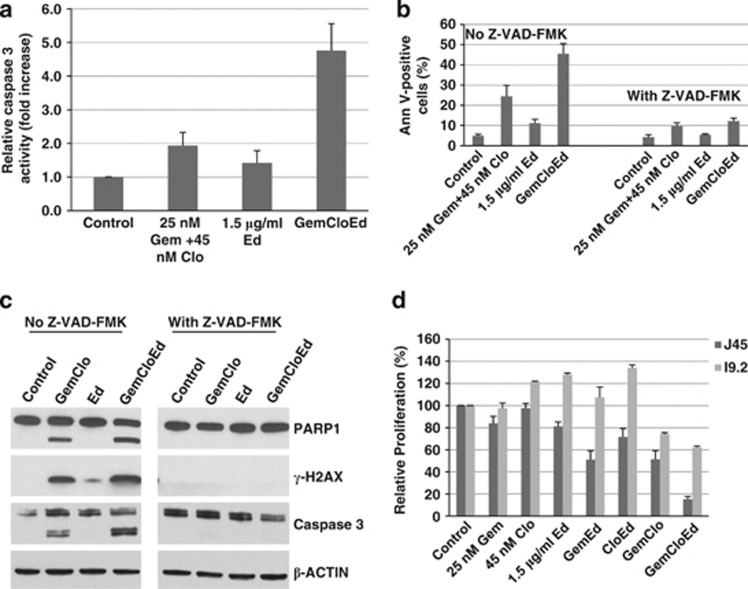
Importance of caspases in the cytotoxicity of [Gem+Clo], Ed and combinations thereof. (**a**) J45.01 cells were continuously exposed to drugs for 48 h and total cell lysates were assayed for caspase 3 activity. To inhibit caspases, cells were exposed to drugs in the presence or absence of 40 μM Z-VAD-FMK, a pan-caspase inhibitor, and cell-death responses were determined by Annexin V assay (**b**) and western blot analysis (**c**). The effects of the three drugs alone or in combination on the proliferation of J45.01 (*Casp 8-*positive) and I9.2 (*Casp 8*-null mutant) cells were compared by exposing cells to drugs for 48 h and determining proliferation by the MTT assay (**d**).

To further analyze the importance of caspases in [Gem+Clo+Ed]-mediated cell death, we determined the enzymatic activity of caspase 3 in drug-treated J45.01 cells. Exposure to [Gem+Clo] combination or Ed alone resulted in a 1.5- to 2-fold increase in caspase activity relative to the control cells while [Gem+Clo+Ed] combination increased it by ∼5-fold (Figure 3a). Pharmacologic inhibition of caspases using Z-VAD-FMK inhibited [Gem+Clo+Ed]-mediated cell death as suggested by the low percentage of Annexin V-positive cells, cleavage of PARP1 and caspase 8, and phosphorylation of histone 2AX (Figures 3b and c). These results are consistent with the observed higher resistance of the Jurkat-derived caspase 8-negative cell line I9.2 to [Gem+Clo+Ed] cytotoxicity when compared with J45.01 cells (Figure 3d).

### [Gem+Clo+Ed] combination activates the production of ROS and decreases mitochondrial membrane potential (Δψm)

**Figure 4:**
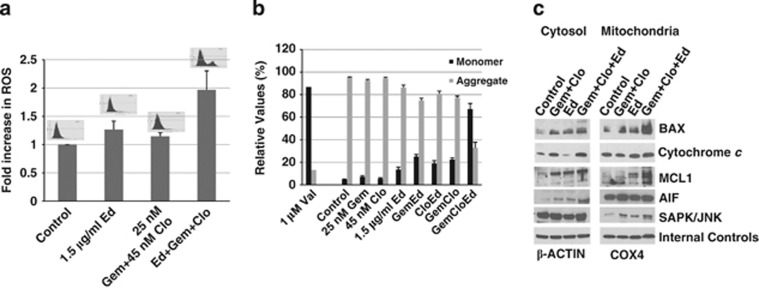
[Gem+Clo+Ed] combination increases the level of ROS and decreases the mitochondrial membrane potential (Δψm). (**a**) J45.01 cells were exposed to drugs for 24 h and early production of ROS was determined using CM-H_2_DCFDA and flow cytometry as described under Materials and Methods. Representative histograms are shown above the bar graphs. (**b**) Changes in Δψm were determined in cells exposed to drugs for 48 h using the JC-1 assay and flow cytometry. Monomers of JC-1 indicate leakage of the mitochondrial membrane and a decreased Δψm while aggregates of JC-1 indicate an intact mitochondrial membrane and high Δψm. (**c**) Cytosolic and mitochondrial fractions were isolated from cells exposed to drugs for 48 h and analyzed by western blotting. β-ACTIN and COX4 were used as an internal control for cytosol and mitochondrial fraction, respectively.

To better understand the cellular responses underlying the [Gem+Clo+Ed]-mediated cell death, we examined the production of ROS, which are known cell-death mediators. Exposure of J45.01 cells to [Gem+Clo] or Ed alone slightly increased the production of ROS relative to the control, while [Gem+Clo+Ed] combination increased it by ∼2-fold (Figure 4a). The results suggest that the three-drug combination has perturbed the mitochondria and increased ROS production. The effects of these drugs on the integrity of the mitochondria are further substantiated by a decrease in the Δψm, which is known to cause leakage of the mitochondrial membrane and release of pro-apoptotic proteins into the cytoplasm.^27^ The decrease in Δψm was determined using JC-1 reagent as previously described.^1^ The aggregated form of JC-1 in the mitochondria emits a red fluorescence and a decrease in Δψm causes translocation of JC-1 reagent to the cytoplasm, where it is converted into its monomeric form that emits a green fluorescence. For example, the control untreated cells showed 95% aggregate and 5% monomer, suggesting retention of JC-1 in the mitochondria due to a high Δψm. As a positive control, J45.01 cells were exposed to valinomycin, a drug that causes permeabilization of mitochondria. Approximately 87% monomer and 13% aggregate form were observed, suggesting a significant decrease in Δψm. Exposure of J45.01 cells to an individual drug resulted in negligible change in JC-1 monomer; exposure to two-drug combinations increased the monomer levels to a maximum of 25% (Figure 4b). A significant decrease in Δψm was observed when cells were exposed to [Gem+Clo+Ed] combination as indicated by 67% JC-1 monomer (Figure 4b), suggesting that [Gem+Clo+Ed] exposure generated stimuli that induced permeability of the mitochondrial membrane.

Consistent with this observation is an increase in the level of pro-apoptotic BAX in the mitochondria of cells exposed to [Gem+Clo+Ed] (Figure 4c). It is believed that BAX forms ion channels and open pores in the mitochondrial membrane which allows the release of pro-apoptotic factors.^28^ Indeed, an increase in the level of cytochrome *c* and apoptosis-inducing factor in the cytoplasm of cells exposed to [Gem+Clo+Ed] was observed (Figure 4c). The increased cleavage of anti-apoptotic MCL1 in the mitochondria is also consistent with [Gem+Clo+Ed]-mediated apoptosis (Figure 4c).

The presence of [Gem+Clo+Ed] might have subjected J45.01 cells to various forms of stress. One stress signal transduction mechanism involves SAPK/JNK, which transmits stress signals into pro-apoptotic events^29^ involving mitochondria.^30, 31^ We, therefore, determined drug-induced localization of SAPK/JNK into mitochondria in J45.01 cells. Figure 4c shows a modest increase in the level of SAPK/JNK protein in the mitochondria when cells were exposed to [Gem+Clo] or Ed; however, a greater increase was observed in cells exposed to [Gem+Clo+Ed]. The drug-mediated activation of the SAPK/JNK pathway and its implications are further discussed below.

### Ed-mediated inhibition of AKT is enhanced by addition of Gem

**Figure 5:**
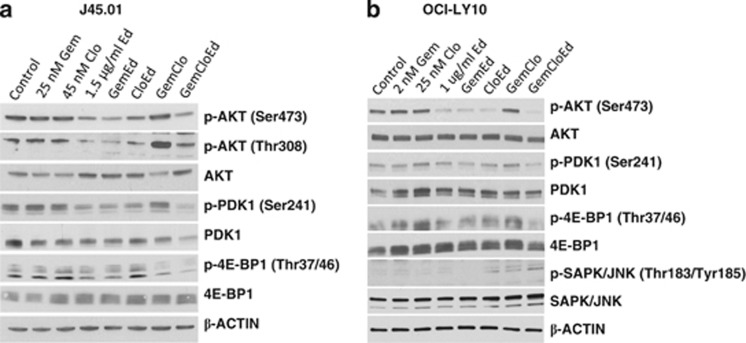
Inhibition of the AKT pro-survival pathway by [Gem+Clo+Ed]. (**a**) J45.01 and (**b**) OCI-LY10 cells were continuously exposed to drugs alone or in combination for 48 h and analyzed by western blotting using the indicated antibodies.

Ed is known to inhibit the pro-survival AKT signaling pathway via AKT dephosphorylation through reorganization of the lipid raft.^32^ We, therefore, determined whether addition of nucleoside analogs would enhance this effect in J45.01 cells. Figure 5a shows that Gem or Clo alone had minimal effect on the level of p-AKT; Ed alone decreased the phosphorylation of AKT at Ser473, a modification catalyzed by the Rictor–mTOR (TORC2) complex and critical for its enzymatic activity.^33^ Exposure of cells to [Gem+Ed] combination further decreased p-AKT levels; a similar decrease was observed with [Gem+Clo+Ed] combination. The results suggest that Gem, but not Clo, increases Ed-mediated inhibition of AKT phosphorylation at Ser473. Interestingly, the level of pan AKT was slightly upregulated when cells were exposed to Ed, with or without nucleoside analogs (Figure 5a).

AKT is also activated by phosphorylation at Thr308 by PDK1.^34^ Since exposure to [Gem+Clo+Ed] combination results in decreased level of p-AKT (Ser473), as discussed above, we sought to determine whether the same combination would also decrease the level of p-AKT (Thr308). Figure 5a shows that Gem or Clo alone did not significantly alter the level of p-AKT (Thr308) but Ed alone, [Gem+Ed] and [Clo+Ed] all decreased p-AKT (Thr308). Interestingly, the combination of Gem and Clo greatly increased the phosphorylation of AKT at Thr308 and addition of Ed reversed this effect (Figure 5a). These results correlate with the observed changes in the activation by phosphorylation of PDK1. The level of p-PDK1 (Ser241) decreased following all drug exposures that included Ed (Figure 5a). Overall, these results suggest that the Ed-mediated decrease in the phosphorylation of AKT at Thr308 and Ser473 may have inhibited the AKT survival pathway and contributed to the cytotoxicity of [Gem+Clo+Ed] in J45.01 cells.

One of the downstream targets of AKT is the Raptor–mTOR (TORC1) complex, which phosphorylates 4E-BP1. A decrease in the activity of AKT is expected to concomitantly decrease the activity of TORC1 and the level of the TORC1 kinase product p-4E-BP1 (Thr37/46). Indeed, a significant decrease in p-4E-BP1 was observed in J45.01 cells exposed to [Gem+Clo+Ed] (Figure 5a), further suggesting a downregulation of the AKT-TORC1 pathway.

To determine whether the drug-mediated repression of the AKT pathway was restricted to the J45.01 T-lymphoblast cell line, we exposed the OCI-LY10 B-cell lymphoma line to similar drug combinations and analyzed the changes in the level of expression and modification of the above-mentioned proteins. The phosphorylation of AKT at Ser473 was similarly inhibited in the presence of Ed, with or without nucleoside analog(s) (Figure 5b). The strongest inhibition was obtained when cells were exposed to [Gem+Clo+Ed] (last lane, Figure 5b). The phosphorylation of both PDK1 and 4E-BP1 was similarly inhibited by the three-drug combination, suggesting equivalent inhibitory effects of [Gem+Clo+Ed] on the AKT pathway in both T-cell and B-cell lymphoma cell lines. Essentially, the same behavior was seen with the second T-cell lymphoma line, SUP-T1 (data not shown).

### [Gem+Clo+Ed] combination activates the SAPK/JNK pathway

**Figure 6:**
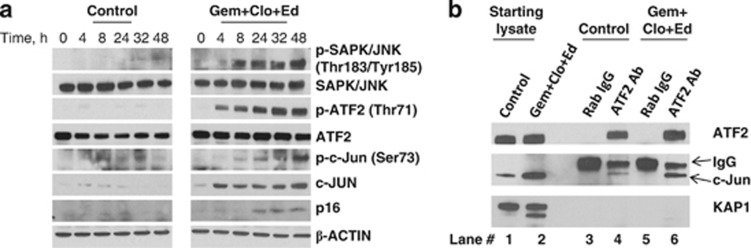
[Gem+Clo+Ed] combination activates the JNK-ATF2 stress signaling pathway. (**a**) J45.01 cells were exposed to solvent (Control) or drugs (Gem+Clo+Ed), harvested at the indicated time point, and analyzed by western blotting. (**b**) Immunoprecipitation assay was performed to determine the interaction of ATF2 and c-Jun in cells exposed to [Gem+Clo+Ed] combination for 48 h. Normal rabbit IgG (Rab IgG) was used to show specificity of the anti-ATF2 antibody.

The observed perturbation of mitochondria in cells exposed to [Gem+Clo+Ed] suggests drug-mediated activation of stress pathways that promote apoptosis. We, therefore, sought to determine the effects of this drug combination on the activation of the SAPK/JNK signal transduction pathway, which is known to transmit and convert stress signaling into apoptosis signaling in various cell types.^31^ Determination of the kinetics of phosphorylation of SAPK/JNK in J45.01 cells exposed to [Gem+Clo+Ed] shows a slight increase in the level of p-SAPK/JNK at Thr183/Tyr185 by 4 h that started to peak after 8 h of drug exposure, with the highest levels being reached after 48 h (Figure 6a). In contrast, there was negligible phosphorylation of SAPK/JNK at Thr183/Tyr185 in the control cells within 48 h (Figure 6a). A similar increase in the phosphorylation of SAPK/JNK at Thr183/Tyr185 was observed in the OCI-LY10 B-cell lymphoma line (Figure 5b) and in the SUP-T1 T-cell lymphoma line (data not shown) after exposure to [Gem+Clo+Ed] for 48 h.

To further dissect the role of the SAPK/JNK signaling pathway in the [Gem+Clo+Ed]-mediated cytotoxicity in J45.01 cells, we analyzed the level of phosphorylation at Thr71 of ATF2, a known substrate for SAPK/JNK.^35^ The kinetics of phosphorylation of ATF2 at Thr71 correlates with the changes in the level of p-SAPK/JNK (Thr183/Tyr185), which starts within 4 h of drug exposure (Figure 6a). A progressive increase was observed up to 32 h when it reached its maximum level. In contrast, negligible p-ATF2 was observed in the control cells.

The phosphorylation of ATF2 is important for its transcriptional activity^36^ and formation of heterodimers with c-Jun.^37, 38^ We, therefore, determined whether c-Jun expression would change in cells exposed to [Gem+Clo+Ed]. Figure 6a shows an increase in the level of c-Jun and p-c-Jun (Ser73) within ∼8 h after exposure to the three-drug combination which peaked after 48 h. Consistent with the increase in the level of p-ATF2 and p-c-Jun is an increase in the expression of one of their target genes *CDKN2A*,^39^ which encodes for p16, in cells exposed to [Gem+Clo+Ed] (Figure 6a). The observed increase in the level of p16 protein, a cyclin-dependent kinase inhibitor that negatively regulates the cell cycle, suggests that the cytotoxicity of [Gem+Clo+Ed] combination is partly due to inhibition of cell-cycle progression.

To further determine whether the interaction of ATF2 with c-Jun was enhanced in cells exposed to [Gem+Clo+Ed], we performed an immunoprecipitation assay. Lysates from cells exposed to solvent (Control) and [Gem+Clo+Ed] combination were prepared. An anti-ATF2 antibody was used to pull down the complex (Figure 6b, lanes 4 and 6) from these lysates; a normal rabbit IgG was used as a negative control (Figure 6b, lanes 3 and 5). Western blot analysis of the immunoprecipitates showed the presence of ATF2 protein in both starting lysates (Figure 6b, lanes 1 and 2) that was pulled down by anti-ATF2 antibody (lanes 4 and 6) but not by normal rabbit IgG (lanes 3 and 5). When the same membrane was probed with anti-c-Jun antibody, signals were obtained in the starting lysates. Again, the level of c-Jun was greater in cells exposed to [Gem+Clo+Ed] relative to the control, consistent with Figure 6a. The anti-ATF2 immunoprecipitates also showed the presence of c-Jun (lanes 4 and 6) that was not seen in the rabbit IgG immunoprecipitates (lanes 3 and 5), suggesting the presence of ATF2 and c-Jun in the same complex. To confirm the specificity of the immunoprecipitation assay, the membrane was probed with an antibody against KAP1, another nuclear protein. The starting lysates (Figure 6b, lanes 1 and 2) showed the presence of KAP1 consistent with Figure 2a, but it was not pulled down with anti-ATF2 antibody, suggesting lack of interaction between KAP1 and ATF2. The absence of KAP1 in the immunoprecipitates suggests that the interaction between ATF2 and c-Jun is specific, which is consistent with previous reports.^37, 38^

## Discussion

**Figure 7:**
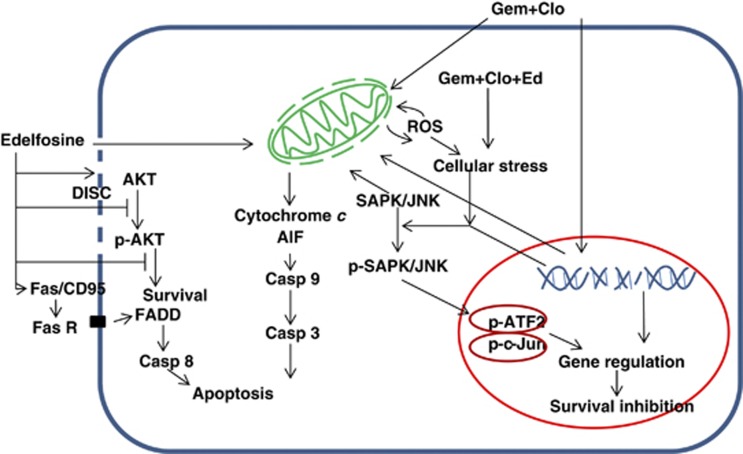
Suggested mechanisms of synergistic cytotoxicity of [Gem+Clo+Ed] combination in lymphoma cells based on the previously reported data and our present results. Edelfosine accumulates in lipid rafts in the cell membrane, leads to formation of death-inducing signaling complex (DISC), activates the extrinsic apoptotic pathway through FADD and caspase (Casp) 8, and inhibits the AKT pro-survival pathway. Edelfosine also decreases the mitochondrial membrane potential leading to activation of the intrinsic apoptotic pathway through the release of pro-apoptotic factors from the mitochondria and activation of caspases 9 and 3. [Gem+Clo] combination, on the other hand, inhibits DNA synthesis and repair leading to a DNA-damage response and apoptosis via the intrinsic pathway. The combination of [Gem+Clo+Ed] magnifies the decrease in the mitochondrial membrane potential, production of ROS and activation of apoptosis. Furthermore, drug-mediated activation of the SAPK/JNK signal transduction pathway through upregulation of ATF2 and c-Jun leads to inhibition of survival.

We recently demonstrated the anti-neoplastic activity of [Gem+Clo] in multiple myeloma cell lines and cells derived from multiple myeloma patients.^1^ We report in the present study strong synergistic cytotoxicity of these two nucleoside analogs when combined with the alkyl phospholipid Ed in lymphoma cell lines of both T- and B-cell origins. This synergism might be attributed to two different mechanisms of cytotoxicity which converge at the disruption of mitochondria, leading to cell death (Figure 7). Edelfosine accumulates in the cell membrane and induces apoptosis by recruitment of Fas/CD95 and subsequent death-inducing signaling complex formation in lipid rafts.^40^ On the other hand, Gem and Clo are nucleoside analogs that mainly target the nucleus and inhibit DNA synthesis and repair, and induce apoptosis through the DNA-damage response.^1^

The cytotoxicity of [Gem+Clo+Ed] combination might be through direct or indirect disruption of the mitochondrial functions. Although Ed associates with the cell membrane, it has also been shown to accumulate in the mitochondria^41^ and it subsequently disrupts the mitochondrial membrane potential (Δψm) leading to apoptosis of leukemic cells.^42^ On the other hand, Gem is phosphorylated by thymidine kinase in the mitochondria,^43^ suggesting that it might inhibit mitochondrial DNA synthesis. The direct binding of Clo triphosphate metabolites to proteins in the mitochondrial membrane is known to decrease Δψm and allow release of pro-apoptotic factors, which are important in the formation of the apoptosome.^44^ Together, these mechanisms may synergistically induce permeabilization of mitochondria leading to a significant decrease in Δψm and activation of caspases (Figures 3, 4 and 7).

The indirect effects of [Gem+Clo+Ed] combination on the mitochondria include the activation of the SAPK/JNK signal transduction pathway, which has previously been shown to induce apoptosis.^31^ This pathway activates receptor-mediated apoptosis in T lymphocytes^45^ and phosphorylates mitochondrial proteins involved in the release of cytochrome *c* and apoptosis-inducing factor.^31^ Our data show activation by phosphorylation of SAPK/JNK as early as 4 h after exposure to [Gem+Clo+Ed] and its accumulation in the mitochondria (Figures 6a and 4c). Our results also suggest that the drug-mediated activation of SAPK/JNK leads to phosphorylation of the transcription factor ATF2, which heterodimerizes with c-Jun. The dimer is known to upregulate expression of pro-apoptotic proteins including caspase 3 and DP5.^46, 47^ The ATF2:c-Jun dimer also activates the expression of c-Jun,^48^ suggesting a positive feedback loop and possible potentiation of the apoptosis signaling pathway through increased expression of caspase 3 and DP5. How [Gem+Clo+Ed] combination activates SAPK/JNK remains to be determined. Since the three-drug combination strongly activates the DNA-damage response (Figure 2a), it is possible that damaged DNA may trigger the phosphorylation of SAPK/JNK consistent with the previously reported activation of the SAPK/JNK-ATF2 pathway by DNA-damaging agents such as cisplatin, actinomycin D, mitomycin C and etoposide.^49, 50^

Activation of the SAPK/JNK stress signaling pathway by [Gem+Clo+Ed] occurs concomitantly with the inhibition of the AKT survival pathway. The kinase activity of AKT depends on its phosphorylation at amino-acid residues Thr308 and Ser473. Our results show inhibition of phosphorylation of these two residues in the presence of Ed (Figure 5). AKT promotes cell survival through phosphorylation and inactivation of pro-apoptotic proteins including pro-caspase 9^51^ and BAD.^52^ Inactivation of the AKT pathway may, therefore, promote apoptosis. Together with the activation of the SAPK/JNK stress signaling pathway, the inhibition of the AKT pathway may partly explain the synergistic cytotoxicity of [Gem+Clo+Ed] toward the J45.01 T-lymphoblast cell line. A similar pattern of concomitant SAPK/JNK activation and AKT inhibition was seen in the OCI-LY10 B-cell lymphoma line (Figure 5b) and in the SUP-T1 T-cell lymphoma line (not shown).

In conclusion, our present results and previously reported data suggest that the synergistic cytotoxicity of [Gem+Clo+Ed] might be due to death-inducing signaling complex formation in lipid rafts, induction of the DNA-damage response, ROS production, destruction of the integrity of mitochondria, inhibition of the pro-survival AKT pathway and activation of the pro-apoptotic SAPK/JNK stress signaling pathway. Together, these mechanisms commit the cells to undergo apoptosis. That such mechanisms are seen in three very different cell lines provides a powerful tool to kill tumor cells and justifies the evaluation of [Gem+Clo+Ed] as a treatment option for lymphoma patients. These findings provided the foundation for us to propose a clinical trial to evaluate this drug combination as a cytoreductive treatment program for patients with lymphomas. Our hypothesis is that, in combination with DNA alkylating agent(s), [Gem+Clo+Ed] will show efficacy when used as a part of the pre-conditioning regimen for lymphoma patients undergoing hematopoietic stem cell transplantation.
